# Benefit-finding in children with advanced cancer and their parents

**DOI:** 10.1017/S1478951524001585

**Published:** 2024-11-15

**Authors:** Emma Siefring, Anna L. Olsavsky, Megan Schaefer, Malcolm Sutherland-Foggio, Alexandra C. Himelhoch, Kylie N. Hill, Ansley E. Kenney, Lisa Humphrey, Randal Olshefski, Cynthia A. Gerhardt

**Affiliations:** 1 Department of Psychology, The Ohio State University, Columbus, OH, USA; 2 Center for Biobehavioral Health, The Abigail Wexner Research Institute at Nationwide Children’s Hospital, Columbus, OH, USA; 3 Division of Pediatric Psychology and Neuropsychology, Natiowide Children’s Hospital, Columbus, OH, USA; 4 Division of Hospice and Palliative Care, Nationwide Children’s Hospital, Columbus, OH, USA; 5 Department of Pediatrics, The Ohio State University, Columbus, OH, USA; 6 Division of Hematology, Oncology, and Bone Marrow Transplant, Natiowide Children’s Hospital, Columbus, OH, USA

**Keywords:** Benefit-finding, cancer, psycho-oncology, pediatric

## Abstract

**Objectives:**

Although pediatric cancer often causes significant stress for families, most childhood cancer survivors are resilient and do not exhibit severe or lasting psychopathology. Research demonstrates some survivors may report benefit-finding or positive outcomes following this stressful life event. However, considerably less research has included families of children who are unlikely to survive their illness. Thus, this study investigated benefit-finding among parents and their children with advanced cancer, as well as associated demographic and medical factors.

**Methods:**

Families (*N* = 72) of children with advanced cancer (ages 5–25) were recruited from a large pediatric hospital. Advanced cancer was defined as relapsed or refractory disease, an estimated prognosis of <60%, or referral to end-of-life care. Participants completed a demographic survey and the Benefit Finding Scale at enrollment.

**Results:**

Children, mothers, and fathers reported moderate to high benefit-finding scores. Correlations between family members were weak and non-significant. Children reported significantly higher benefit-finding than fathers. Demographic and medical factors were not associated with benefit-finding in children, mothers, or fathers.

**Significance of results:**

Families of children with advanced cancer reported moderate to high benefit-finding regardless of background or medical factors. Children identified benefits of their cancer experience independent of the experiences of their mothers and fathers. Larger studies should continue to examine factors associated with positive and negative outcomes in the context of childhood cancer to inform interventions.

## Introduction

Childhood cancer is the leading cause of death by illness for children in the United States (Siegel et al. 2019). Each year, over 20,000 children under the age 20 are newly diagnosed with cancer, and 1,800 will die from their illness (Siegel et al. 2019). As treatments have improved over the years, survival rates have also increased, with 83.4% of children now living for at least 5 years following diagnosis, including those with advanced cancer and likely incurable disease (Siegel et al. 2019). Children with advanced cancer can experience a protracted cycle of treatments, relapses, and remissions and/or extended periods of refractory or quiescent disease. To date, little research has focused on the unique experiences of children with advanced cancer.

Given the stress associated with pediatric cancer, children and their parents are at increased risk for psychological distress. Children with cancer and adult survivors of pediatric cancer may have greater symptoms of anxiety, depression, posttraumatic stress (PTSS), and lower quality of life compared to healthy counterparts (Krull et al. 2008; Wulff-Burchfield et al. 2019; Zeltzer et al. 2009). Children with leukemia or cancers affecting the central nervous system (CNS) are at an even higher risk for psychosocial difficulties (Krull et al. 2008; Wulff-Burchfield et al. 2019; Zeltzer et al. 2009) due to the neuro-toxic treatments that may result in cognitive impairments and social and emotional challenges (Krull et al. 2008; Wulff-Burchfield et al. 2019; Zeltzer et al. 2009). Furthermore, parents of children with cancer are also at risk for higher levels of anxiety, depression, and PTSS (Clarke et al. 2009; Klassen et al. 2007; Pai et al. 2007; Jantien Vrijmoet-Wiersma et al. 2008). Notably, parents of children nearing end-of-life may experience higher rates of PTSS and poorer adjustment when compared to parents of childhood cancer survivors (Rosenberg et al. 2012).

Although a subset of parents and children with cancer struggle to adjust to the illness, many individuals are resilient and do not exhibit severe or lasting psychological distress (Stuber and Strom 2012). Positive psychology approaches shift away from focusing only on psychological impairment, with greater attention to adaptive functioning (Gable and Haidt 2005). Benefit-finding, defined as identifying positive outcomes in relation to a stressful life event, is an example of a positive psychology construct (Helgeson et al. 2006). While posttraumatic growth requires one to experience a traumatic event, benefit-finding can occur in relation to a stressful, but not necessarily traumatic, event and can happen regardless of whether someone identifies a fundamental shift in their life perspective (Gardner et al. 2017b; Phipps et al. 2007). Both children with cancer and their parents have been found to report benefit-finding in response to the child’s cancer diagnosis and treatment (Michel et al. 2010). To our knowledge, research has yet to examine associations between child and parent benefit-finding, as well as associated factors, particularly in the context of advanced pediatric cancer (Michel et al. 2010).

Demographic factors have been associated with benefit-finding and posttraumatic growth among children with cancer. In regards to age, some studies have found that older children with serious illnesses report more benefit and growth (Barakat et al. 2006; Helgeson et al. 2006; Husson et al. 2017; von Rezori et al. 2022), while 1 study found no association between age and benefit-finding (Phipps et al. 2007). Data are mixed on sex differences in benefit-finding, with some studies finding similar levels between male and female children with cancer (Michel et al. 2010; Phipps et al. 2007), while others note greater posttraumatic growth (Husson et al. 2017), and benefit-finding in females (von Rezori et al. 2022). With respect to race, some studies have not identified differences in levels of posttraumatic growth based on race (Cordova et al. 2001; Husson et al. 2017), but others have demonstrated that BIPOC (Black, Indigenous, people of color) children report increased benefit-finding compared to their White counterparts (Conley et al. 2020; Helgeson et al. 2006; Phipps et al. 2007). Lastly, some research among adults and families with cancer have found no correlation between income and benefit-finding (Barakat et al. 2006; Lechner et al. 2003), while 1 study demonstrated that higher income correlated with higher posttraumatic growth among adults with cancer (Cordova et al. 2001).

In addition to demographics, the role of medical factors in benefit-finding and posttraumatic growth has been investigated. Time since diagnosis was correlated with higher levels of benefit-finding in a study with adult survivors of breast cancer (Cordova et al. 2001), while other studies have found no association between time since diagnosis and posttraumatic growth (Pakenham and Cox 2009; Stanton et al. 2006). Another study found that greater time since diagnosis was negatively correlated with benefit finding (Prikken et al. 2022). Type of diagnosis may be important due to the impact of various treatments on quality of life. One study demonstrated that survivors of leukemia and CNS cancers experienced more benefit-finding compared to survivors of solid tumors (Michel et al. 2010), with another study finding that more intensive cancer treatments correlated with more benefit finding (Prikken et al. 2022).

Most research has examined benefit-finding in adults with cancer, while few studies have included children with cancer. Of these studies, most occur following treatment completion or have a heterogenous sample, including children who were either newly diagnosed or were many years past diagnosis (Klassen et al. 2007). To date, only 1 study has examined benefit-finding in children with advanced cancer and their parents (Schaefer et al. 2021). Schaefer and colleagues qualitatively explored meaning-making in children with advanced cancer and their parents, in which benefit-finding was identified as a major theme. In this study, children with advanced cancer identified benefits such as strengthened relationships with loved ones and a greater appreciation for life (Schaefer et al. 2021). The authors (Schaefer et al. 2021) note that the use of quantitative measures of benefit-finding are needed to further understand the experiences of individuals near the end-of-life to better inform interventions targeting the prevention or reduction of psychological suffering (Hinds et al. 2004).

Thus, this study aims to quantitatively characterize benefit-finding among children with advanced cancer and their parents and to identify individual and medical factors related to benefit-finding. This study fills a gap in the literature by examining benefit-finding in children with advanced cancer and exploring the association between parent and child benefit-finding. Based on the literature described above, we expected that child and parent benefit-finding would be positively correlated, but that parents would have significantly greater benefit-finding than children. We also predicted that older age, female sex, greater time since diagnosis, and a CNS tumor diagnosis would be related to higher levels of benefit-finding in children with advanced cancer.

## Methods

Data are part of a larger study examining quality of life and decision-making in children with advanced cancer and their parents (Siefring et al. 2018). The larger study involved assessments and interviews with families across 3 time-points: enrollment (i.e., T1), 6 months, and 12 months, as well as monthly, brief online surveys regarding the child’s symptoms and quality of life.

### Participants

Parents and children were eligible for the study if the child had the following characteristics: advanced cancer (defined as any relapsed or refractory disease, physician-estimated survival <60%, or referral to end-of-life care), aged 5–25 years old, had at least 1 parent who spoke English, and lived within 140 miles of the hospital. Children with significant developmental disabilities were not eligible.

### Sample characteristics

Of 149 eligible families, 72 (48.3%) enrolled in the study, resulting in 56 mothers, 30 fathers, and 49 children. Two mothers were excluded due to incomplete data. Please refer to Table 1 for demographics. Of the 72 families that participated in the study, 23 children did not self-report data because they were either too ill too young. Of the 49 children who provided self-report, the average age was 14.4 years old (*SD* = 3.82). Of all 72 children, including those who did not self-report, the average age at T1 was 12.6 years old (*SD* = 4.80), and most were male (*n* = 42; 58.3%) and White (*n* = 58; 80.6%). The most common diagnosis was a solid tumor (*n* = 30; 41.7%) with an average age at diagnosis of 10.1 years old (*SD* = 5.18).

**Table 1.** Demographic characteristics of mothers, fathers, and all children

	Mothers	Fathers	Children
	(*n* = 55)	(*n* = 30)	(*n* = 72)
***M (SD)***			
Age at T1 (yrs)	40.73 (6.70)	43.59 (6.67)	12.6 (4.8)
Age at diagnosis (yrs)			10.1 (5.2)
Time since diagnosis (mo)			31.3 (40.1)
	***n (%)***		
Education completed			
High school or grade school	13 (23.6%)	7 (23.3%)	
College, technical/trade school	28 (50.9%)	16 (53.3%)	
Graduate/ professional degree	13 (23.6%)	7 (23.3%)	
Unknown	1 (1.8%)		
Income			
Under-$25,000 per yr	13 (25.0%)	6 (20.7%)	
$25,001–$50,000 per yr	10 (19.2%)	3 (10.3%)	
$50,000–$75,000 per yr	7 (13.5%)	6 (20.7%)	
$75,001–$100,000 per yr	7 (13.5%)	6 (20.7%)	
$100,001–$150,000 per yr	5 (11.5%)	5 (17.2%)	
$150,001 or more	9 (17.3%)	3 (10.3%)	
Unknown	3 (4.2%)	1 (4.2%)	
Race			
Black/African-American	1 (1.8%)	1 (3.3%)	2 (2.8%)
American Indian/Native Alaskan	0 (0%)	0 (0%)	0 (0%)
Asian	4 (7.3%)	3 (10.0%)	4 (5.6%)
Native Hawaiian/Pacific Islander	0 (0%)	0 (0%)	0 (0%)
Race not listed	5 (9.1%)	1 (3.3%)	7 (9.7%)
White	45 (81.8%)	25 (83.3%)	58 (81.7%)
Ethnicity			
Non-Hispanic/Latinx	54 (98.1%)	30 (100%)	72 (100%)
Hispanic/Latinx	0 (0%)	0 (0%)	0 (0%)
Unknown	1 (1.8%)	0 (0%)	0 (0%)
Sex			
Male	0 (0%)	30 (100%)	42 (58.3%)
Female	55 (100%)	0 (0%)	30 (41.7%)
Diagnosis			
Leukemia			21 (29.2%)
Lymphoma			6 (8.3%)
Brain tumor			15 (20.8%)
Other solid tumor			30 (41.7%)

### Procedures

Institutional Review Board approval (IRB16-00869) was obtained at a children’s hospital in the Midwestern United States. Eligible families were identified through the palliative care or oncology teams, as well as through a review of inpatient records. The study coordinator contacted families in the clinic, hospital, or via phone to introduce the study and assess interest in participation. Following informed consent/assent, study staff scheduled a time to conduct an initial assessment with parents and children in the hospital, virtually, or at their home. Children ≥8 years old completed assessments if they were able to provide self-report. Only parent report was obtained for children aged 5–7 years old. Research assistants conducted individual assessments with participants, either virtually or in-person, depending on their enrollment during the COVID-19 pandemic, to ensure confidentiality and provide assistance as needed. Families were compensated $40 for the initial home/hospital visit and $5 for monthly measurements.

### Measures

### Demographic questionnaire

A demographic form was created by the research team to collect family background information such as age, date of birth, education level, race, ethnicity, household income, and religion.

#### The Benefit Finding Scale (BFS)

The BFS was used in this study to measure benefit-finding in parents across all 3 time-points (Antoni et al. 2001; Gardner et al. 2017a). This measure includes 17 items and assesses areas such as personal growth, relationship improvement, and purpose in life (e.g., brought my family closer together; helped me become a stronger person). Items were rated from 1 “not at all” to 5 “extremely,” resulting in an average item score. Higher scores indicated higher levels of benefit-finding. Evidence supports internal reliability, convergent and discriminant validity (Antoni et al. 2001), and construct validity (Li et al. 2017; Pascoe and Edvardsson 2015). Internal consistency was acceptable for mothers (*α* = .93) and fathers in this sample (*α* = .93).

#### The Benefit Finding Scale for Children (BFSC)

This instrument was adapted, with minor rewording, from the adult BFS to assess benefit-finding in children (Phipps et al. 2007). Ten items (e.g., brought my family closer together; helped me become a stronger person) are rated from 1 “not at all true for me” to 5 “very true for me,” resulting in an average item score. Higher scores indicated higher levels of benefit-finding. The BFSC has excellent reliability (Michel et al. 2010; Phipps et al. 2007). Children were asked to complete this measure at all 3 time-points. Internal consistency was acceptable in this sample (*α* = .84).

#### Electronic medical records

Medical records were reviewed by research staff using a structured form to collect data about diagnosis, time since diagnosis, and treatment.

### Analysis plan

Descriptive statistics were calculated for variables of interest. Average item scores were calculated only when at least 80% of items on the measure were completed by participants. Associations between parent and child benefit-finding were examined using Pearson correlations (*α* = .05, 2-way), and paired *t*-tests (*α* = .05, 2-way) were used to examine differences between informants within families (i.e., mother–child, father–child, mother–father). While the BFS and BFSC have a different number of items, the 2 can be directly compared using average item scores as the constructs for both are presumed to be the same and adapted from the same measure. Associations between medical/demographic factors and benefit-finding in both parents and children were also examined using Pearson correlations or *t*-tests (*α* = .05, 2-way) as appropriate. Cohen’s *d* effect sizes were calculated for paired comparisons. To determine the relative contributions of significant demographic or medical factors to child and parent benefit-finding, hierarchical regressions were planned. Using Gpower, the sample of 54 mothers provided power (.66–.82) to detect medium effects for *t*-tests (*d* = .50) and correlations (*r* = .30) (Faul et al. 2007). The sample of 30 fathers and 49 children provided power (.83–.97) to detect large effects for *t*-tests (*d* = .80) and correlations (*r* = .50), respectively.

## Results

### Benefit-finding in mothers, fathers, and children

The average benefit-finding score for mothers was 3.71 (*SD* = .81), while the average for fathers was 3.12 (*SD* = .84). In children, the average benefit-finding score was 3.84 (*SD* = .74), indicating moderate to high benefit-finding. Correlations are included in Table 2. Child and mother benefit-finding scores did not show a significant correlation, *r*(33) = .16, *p* *=* .36. Child and father benefit-finding scores showed a moderate correlation that was non-significant, *r*(19) = .33, *p* *=* .17. Mother and father benefit-finding scores were moderately correlated but also not significantly different, *r*(19) = .36, *p* *=* .13. Paired sample *t*-tests revealed there were no significant differences between mother (*M* = 3.64, *SD* = .85) and child benefit-finding scores (*M* *=* 3.46, *SD* = 1.08), *t*(32) = .84, *p* = .41; *d* = .19. However, father benefit-finding scores (*M* = 3.10, *SD* = .91) were significantly lower than child scores (*M* *=* 3.94, *SD* = .75), *t*(18) = −3.78, *p* = .001; *d* = 1.01. Mother (*M* = 3.64, *SD* = .83) and father (*M* = 3.23, *SD* = .81) benefit-finding scores did not significantly differ from one another, *t*(18) = 1.97, *p* = .06; *d* = .50.

**Table 2.** Correlations between benefit-finding and demographic variables

	Child BFSC	Mother BFS	Father BFS
Child BFS	–	–	–
Mother BFS	.16	–	–
Father BFS	.33	.36	–
Child age at T1	.15	−.10	−.29
Mother age at T1	.12	−.02	−.37
Father age at T1	−.06	−.15	−.30
Child age at diagnosis	.20	.03	−.29
Child time since diagnosis	−.09	−.16	.00
Mother education	−.11	.06	.15
Father education	−.11	−.04	.02
Mother income	−.07	−.07	−.07
Father income	−.18	−.19	.01

Note: *N* ranges from 17 to 54. All correlations non-significant.

### Associations between benefit-finding, demographic characteristics, and medical factors

Among children, there were no significant differences in benefit-finding between males (*M* = 3.53, *SD* = .87) and females (*M* = 3.43, *SD* = 1.21); *t*(47) = .32, *p* = .75; *d* = .10. Benefit-finding scores did not differ between White (*M* = 3.54, *SD* = 1.00) and BIPOC children (*M* = 3.21, *SD* *=* 1.07); *t*(47) = .78, *p* = .44; *d* = .32. Child scores were not significantly correlated with age at enrollment, *r*(49) = .15, *p* = .29, child age at diagnosis, *r*(49) = .20, *p* *=* .18, nor time since diagnosis, *r*(49) = −.09, *p* = .54. Due to small sample sizes, leukemia and brain tumor diagnoses were collapsed into 1 group, and lymphoma and other solid tumors were collapsed into a second group based on the risk of treatment affecting the CNS. There were no significant differences in benefit-finding between children diagnosed with leukemia/brain tumors (*M* = 3.40, *SD* = 1.20) versus those diagnosed with lymphoma/other solid tumors (*M* = 3.55, *SD* = .85); *t*(47) = −.51, *p* = .61; *d* = −.14.

Parent age was unrelated to mother, *r*(54) = −.02, *p* *=* .86, and father benefit-finding scores, *r*(30) = −.30, *p* *=* .11. Level of education was also not correlated with mother, *r*(53) = .06, *p* = .70, or father benefit-finding scores, *r*(30) = .02, *p* = .91. With regard to child medical factors, time since diagnosis and age of child at diagnosis were not significantly correlated with mothers’, *r*(53) = −.16, *p* *=* .26 and *r*(53) = .03, *p* *=* .85, or fathers’ benefit-finding scores, *r*(30) = .004, *p* *=* .98 and *r*(30) = −.29, *p* *=* .12, respectively. Benefit-finding scores did not significantly differ between White mothers (*M* = 3.10, *SD* = .80) and BIPOC mothers (*M* = 3.73, *SD* *=* .97); *t*(52) = −.07, *p* = .94; *d* = −.71. Sample size restraints limited comparisons based on race of fathers.

Due to the lack of significant associations between benefit-finding and other demographic and medical factors, planned multi-variate models examining their relative contribution to benefit-finding were not conducted.

## Discussion

Limited research has examined benefit-finding in children with advanced cancer. Using standardized measures with children with advanced cancer and their parents, this study is one of the first to explore benefit-finding among multiple family members, as well as potential associations with demographic and medical factors. Results show that despite having an unlikely prospect of cure, children with advanced cancer and their parents indicated moderate to high levels of benefit-finding from the child’s cancer experience. In general, family members reported benefits independent of one another, and few demographic or medical factors associated with variability in benefit-finding.

In this study, children with advanced cancer reported moderate to high levels of benefit-finding, which was higher than previous studies examining benefit-finding in children with cancer (Michel et al. 2010; Phipps et al. 2007; Prikken et al. 2022). This could be due to the curvilinear relationship between stress and benefit-finding, which proposes that either too little or too much stress will not result in benefit-finding (Cordova et al. 2001; Helgeson et al. 2006; Lechner et al. 2003). Given that most children in the sample were not imminently at the end of life but were navigating a poor prognosis, it is possible that they had sufficient, but not overwhelming, stress to account for this effect. This possibility could be further explored by examining benefit-finding more longitudinally when patients are further along their disease progression and potentially nearing end-of-life.

Parent benefit-finding scores were also moderate to high and comparable to scores in adults with cancer (Applebaum et al. 2020; Lechner et al. 2003; Llewellyn et al. 2013). When compared to child benefit-finding scores, father benefit-finding scores were significantly lower. It is possible that fathers may process the trauma of their child’s cancer diagnosis differently than the child; however, future work is still needed to better understand father–child dyads in pediatric oncology (Stanton et al. 2006). Although fathers had significantly lower benefit-finding scores compared to their children, it should be noted that fathers still identified moderate levels of benefit-finding. Our data also suggest that child and parent benefit-finding appear to be independent of one another. Benefit-finding between father and child, mother and child, and mother and father were not significantly correlated, as was found in a study with childhood cancer survivors (Michel et al. 2010). However, the moderately sized correlations between mothers and fathers in the current study suggest the possibility of associations and need for additional research.

Unlike other studies investigating benefit-finding in individuals with cancer, we did not find significant associations with demographic or medical factors. This highlights that benefit-finding may occur in children and parents regardless of background or medical factors. Most of the literature suggests a curvilinear relationship between age and benefit-finding, with young children and older adults finding less benefit than adolescents and adults (Helgeson et al. 2006; Husson et al. 2017). Our sample did not exhibit significant correlations between age at enrollment and benefit-finding in either parents or children, as previously found in the child literature (Phipps et al. 2007). Child age at diagnosis was also not significantly related to benefit-finding, contrasting with other studies showing that children who are younger at diagnosis find less benefit than children who are older (Barakat et al. 2006; Michel et al. 2010; Phipps et al. 2007). This could be a result of our study criterion allowing only children aged ≥8-year-old to complete self-reports of benefit-finding.

Time since diagnosis also did not exhibit significant associations with benefit-finding, in contrast to other studies that demonstrated the longer time between diagnosis and study completion, the less benefit-finding reported (Michel et al. 2010; Phipps et al. 2007). This is likely because both of these studies recruited cancer survivors who were many years past diagnosis and treatment. However, because our sample of children with advanced cancer were still undergoing treatment during the study, they may have been less likely to show the same decline in benefit-finding, with this as another reason to further explore benefit-finding longitudinally (Phipps et al. 2007).

Other demographic characteristics such as sex, family income, race, ethnicity, parent education, and cancer type did not reveal any significant associations with mother, father, or child benefit-finding. With regard to sex differences, male and female children did not have significantly different benefit-finding scores, which has been found in previous literature (Lechner et al. 2003; Michel et al. 2010; Phipps et al. 2007). There were also no significant differences between mothers and fathers, contrasting the literature that supports sex differences in benefit-finding (Barakat et al. 2006; Helgeson et al. 2006; Husson et al. 2017; Tomich and Helgeson 2004). However, our study had a small number of complete parent dyads, which restricted our ability to compare scores between mothers and fathers.

Child benefit-finding was not significantly different when compared between diagnostic groups, contrasting with research showing that survivors with prior histories of leukemia and CNS tumors found more benefit compared to other diagnosis groups (Michel et al. 2010). Survivors who had leukemia and CNS tumors may have found more benefits from their cancer journey compared to others due to varying aspects of their illness experience (e.g., length of treatment, long-term side effects). In this study, all participants were still receiving some form of treatment, so differences in benefit-finding scores across diagnostic groups may not have been as evident. Of note, we were limited in our comparison of treatment groups, as only 2 children with CNS tumors were able to complete the benefit-finding measure. This may be a result of cognitive impairments that are often associated with extensive treatment for CNS tumors, suggesting a need for further research in this specific population.

### Study limitations

Limitations of this study should be considered when reviewing our conclusions. Restrictions due to the COVID-19 pandemic disrupted recruitment in healthcare centers and disrupted in-person data collection for immunocompromised patients. Despite attempts to shift to remote methods, recruitment slowed from >85% pre-COVID resulting in a smaller sample than anticipated. Participation of each family member also varied. Only 30 fathers from 72 families participated in the study for a variety of reasons (families with 2 mothers, single parent families, etc.). This restricted our ability to examine associations with child and mother benefit-finding, as well as differences based on background factors for fathers. Additionally, comparisons were calculated between the adult measure (BFS) and child measure (BFSC) of benefit-finding. The BFSC is altered to meet the developmental needs of children, thus, interpretation of these comparisons should be done so keeping in mind the differences in measures. The overall sample size also led to a smaller number of children with brain tumors who were able to complete measures. Brain tumors are more likely to be diagnosed in young children (under age 5), which limited enrollment and the ability to compare benefit-finding across diagnosis groups. The sample was recruited from only 1 site and was predominantly White and non-Hispanic. Future studies should include multiple institutions to increase diversity and generalizability to other regions and populations. Given the cross-sectional design, studies should also investigate benefit-finding at multiple time-points over a longer period of time to understand how benefit-finding develops and evolves for both children and parents. Lastly, researchers should investigate how benefit-finding may serve as a protective factor against distress and other negative outcomes.

### Clinical implications

Despite these limitations, this study is one of the first to examine benefit-finding among children with advanced cancer and explore associations between child and parent benefit-finding. Clinicians and parents can be reassured that many children with advanced cancer exhibit resilience amidst significant adversity. Such adversity may bring into sharper focus the importance of close relationships, positive experiences, and finding a silver lining in the midst of unimaginable challenges. Clinicians may consider incorporating meaning-making into psychosocial interventions, which has been shown to be valuable in both children, parents, and adults at end-of-life (Breitbart et al. 2004; Schaefer et al. 2021). However, this intervention should be implemented with caution and acknowledgement that not all patients and families impacted by pediatric cancer will experience benefit-finding throughout their child’s illness journey. We encourage clinicians to monitor benefit-finding language (e.g., “I am closer with my mom now; this experience has put life into perspective; I have learned to not sweat the small things”) and provide behavioral observations (e.g., watching relationships grow, witnessing focus on values-based actions) to highlight benefit-finding for patients and families when appropriate. The term “benefit-finding” may be perceived as minimizing an individual’s struggles, so we suggest avoiding the term or using it with caution. Additionally, clinicians can interpret from this data that parent and child benefit-finding may be independent of one another and can be fostered regardless of how other members of the family experience benefit-finding.

### Conclusions

Children with advanced cancer and their parents can identify benefits from their cancer experience, and demographic and medical factors do not necessarily determine who is more or less likely to find benefits. Thus, children with advanced cancer and their parents can still navigate the positive and meaningful aspects of their lives despite being faced with a pediatric life-threatening illness.
